# A Comprehensive Review of Unobtrusive Biosensing in Intelligent Vehicles: Sensors, Algorithms, and Integration Challenges

**DOI:** 10.3390/bioengineering12060669

**Published:** 2025-06-18

**Authors:** Shiva Maleki Varnosfaderani, Mohd. Rizwan Shaikh, Mohamad Forouzanfar

**Affiliations:** 1 Electrical Engineering Department, Wayne State University, Detroit, MI 48202, USA; shiva.maleki.varnosfaderani@wayne.edu; 2 École de Technologie Supérieure (ÉTS), Université du Québec, Montreal, QC H3C 1K3, Canada; mohdrizwan.shaikh@iiitb.ac.in; 3 International Institute of Information Technology Bangalore, Bengaluru 560100, Karnataka, India; 4 Centre de Recherche de l’Institut Universitaire de Gériatrie de Montréal, Montreal, QC H3W 1W5, Canada

**Keywords:** driver safety enhancement, in-vehicle physiological monitoring, unobtrusive biosensing

## Abstract

Unobtrusive in-vehicle measurement and the monitoring of physiological signals have recently attracted researchers in industry and academia as an innovative approach that can provide valuable information about drivers’ health and status. The main goal is to reduce the number of traffic accidents caused by driver errors by monitoring various physiological parameters and devising appropriate actions to alert the driver or to take control of the vehicle. The research on this topic is in its early stages. While there have been several publications on this topic and industrial prototypes made by car manufacturers, a comprehensive and critical review of the current trends and future directions is missing. This review examines the current research and findings in in-vehicle physiological monitoring and suggests future directions and potential uses. Various physiological sensors, their potential locations, and the results they produce are demonstrated. The main challenges of in-vehicle biosensing, including unobtrusive sensing, vehicle vibration and driver movement cancellation, and privacy management, are discussed, and possible solutions are presented. The paper also reviews the current in-vehicle biosensing prototypes built by car manufacturers and other researchers. The reviewed methods and presented directions provide valuable insights into robust and accurate biosensing within vehicles for researchers in the field.

## 1. Introduction

With the growth of the automotive industry, road traffic accidents have significantly increased, becoming the leading cause of death by injury and the eighth-leading cause of death globally [1]. The World Health Organization (WHO) reports that road traffic accidents cause approximately 1.35 million deaths and up to 50 million non-fatal injuries annually, with many of the injuries resulting in permanent disability. In 2017, the National Highway Traffic Safety Administration (NHTSA) found that driver distraction was reported in about 9% of all fatal crashes in the U.S.—involving 3166 fatalities out of 37,133 total road deaths that year [2]. Distracting activities include, but are not limited to, eating, drinking, and using a phone [3]. Globally, road traffic incidents are the leading cause of death for children and young adults ages 5–29, resulting in approximately 1.19 million deaths per year, a figure comparable to serious diseases like HIV. Additionally, 20 to 50 million more people suffer non-fatal injuries, many of which result in long-term disability [4,5]. According to NHTSA’s “Critical Reasons for Crashes” study, the driver was identified as the critical actor in approximately 94% (±2.2%) of crashes—meaning they were the last to act before the collision—categorized into recognition, decision, performance, and non-performance errors, many of which relate to their physiological or cognitive state [6].

Given that many crashes are linked to the driver’s physiological or cognitive state, continuous monitoring of these states has become increasingly important—especially as vehicles become more automated. Physiological monitoring plays a critical role in this context [7]. While Society of Automotive Engineers (SAE) Level 5 vehicles are fully autonomous, Levels 3 and 4 still require the driver to take control under specific conditions [8,9]. Ensuring that drivers are mentally and physically prepared to resume control is therefore essential for maintaining safety and reducing accident rates [9]. Moreover, the research highlights a phenomenon known as the “irony of automation,” where increased vehicle autonomy leads to decreased driver attention and engagement [10]. As a result, even in semi-autonomous driving, monitoring the driver’s status remains vital [9].

To evaluate a driver’s cognitive and physiological condition, researchers generally use three types of methods: subjective self-assessments, behavioral indicators, and physiological measurements. However, subjective assessments are impractical and unreliable during driving tasks, making objective approaches more suitable [11]. Among these, physiological sensing—especially through unobtrusive wearable or contactless technologies—has emerged as a viable solution. It enables real-time assessment of driver states, such as fatigue, distraction, drowsiness, and stress, all of which are critical factors in traffic safety.

Monitoring a driver’s status through unobtrusive sensors has emerged as an innovative approach to prevent or reduce traffic accidents caused by driver errors [12]. The advancement in wearable and contactless technologies has made it possible to monitor driver status better than before. Various physiological and non-physiological sensors have been, or have the potential to be, adopted to monitor the driver’s status, including stress level [13], fatigue [14], drowsiness and vigilance [15,16], distraction [17], dangerous driving [18], and various health conditions [19,20]. For this purpose, different biosignals, such as electroencephalogram (EEG) [21,22,23], electrooculogram (EOG) [24], electromyogram (EMG) [25], electrocardiogram (ECG) [26], photoplethysmogram (PPG) [27], ballistocardiogram (BCG) [28], electrodermal activity (EDA) [29], skin temperature [30], blood pressure [31], and respiration [32], have been measured and analyzed, along with other measures of driving behavior extracted through cameras and car mechanical sensors.

Research on integrating biosensors into vehicles is in the early stages. While some car manufacturers have developed prototypes, significant progress is needed. Effective biosensing systems must include non-intrusive sensors that accurately monitor physiological signals with minimal contact, withstand environmental noise, and process data in real time to ensure reliable outcomes. Adaptive algorithms that merge data from multiple sensors are crucial to accurately determine physiological parameters despite noise and disruptions.

**Figure 1:**
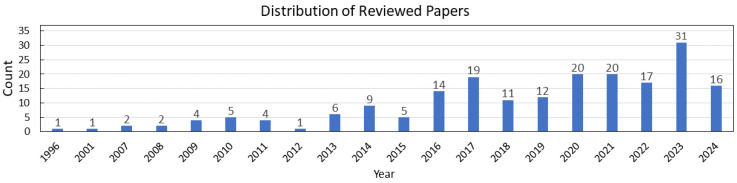
Year-wise distribution of reviewed papers.

This paper presents a comprehensive and up-to-date review of in-vehicle biosensing technologies. We conducted an extensive literature search using platforms like Google Scholar, Web of Science, and IEEE Xplore, with keywords such as “vehicle biosensing,” “physiological monitoring,” “driver health,” and “contactless sensing.” Our review focuses on work published from 2019 onward while also referencing foundational studies where relevant. Figure 1 shows the year-wise distribution of the reviewed publications.

Unlike earlier reviews that focused narrowly on either sensor types or single use cases (e.g., drowsiness detection), our work provides a broader structured comparison across sensing modalities (contact and contactless), physiological targets (e.g., stress, fatigue, and cardiac activity), and signal processing techniques. We also explore the placement of these sensors in the vehicle cabin, practical challenges in deployment, and the integration of biosensing with machine learning for real-time monitoring. Our inclusion of industrial prototypes and commercial developments also bridges the gap between academic research and real-world implementation, which is often underrepresented in earlier literature.

Overall, this review serves as a valuable resource for researchers and practitioners, offering a consolidated foundation for developing next-generation in-vehicle biosensing systems that are adaptive, robust, and practical.

## 2. Integrating Biosensors in Automotive Environments

**Figure 2:**
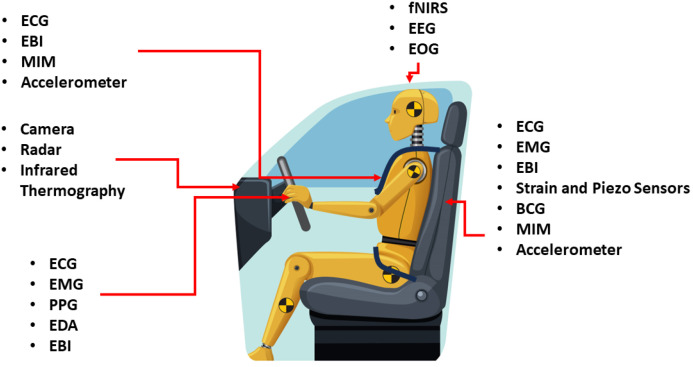
Potential sensor locations in the vehicle.

In-vehicle biosensing can be achieved using various physiological sensors, classified into two categories: contact and contactless. Figure 2 illustrates these sensor types and their potential placements within a vehicle. Contact sensors can be further divided into direct contact and non-direct contact. Direct-contact sensors require direct skin contact to measure physiological parameters. Examples include biopotential sensors capturing bioelectrical activities, electrical sensors measuring changes in electrical properties, and optical sensors using light transmission through the skin. These sensors often need electrodes, cuffs, cables, and pneumatic lines, making them more suited to clinical settings, but they are also explored for automotive use to monitor driver health and safety [33].

Non-direct-contact sensors also require physical contact but not directly with the skin. They can be embedded in objects the driver interacts with, such as a capacitive or textile ECG embedded in the seat, BCG sensors integrated into the seat, or accelerometers attached to the belt or seat.

While contact sensors need consistent contact, which may not be feasible during driving, contactless sensors offer a more practical solution by eliminating the need for direct physical interaction [34,35,36]. However, they may encounter additional noise and artifacts, requiring advanced algorithms to accurately estimate physiological parameters indirectly. This section reviews the application of different sensing modalities to physiological sensing in vehicles, discussing potential challenges, limitations, and possible solutions.

A list of the existing literature on the design of biosensing systems in vehicles based on sensing modality, sensor location, physiological measures, and methodology is presented in Table 1. Most studies focus on sensors mounted on the steering wheel [30,37,38,39,40,41,42], car seat [28,43,44,45,46,47], or seat belt [47,48], which rely on proper contact with the driver. Recent studies have explored contactless technologies like radar [44,49,50] and cameras [51] for in-vehicle biosensing.

Table 1 also summarizes the limitations of the current in-vehicle biosensing techniques. The common issues include the lack of studies on the effects of road noise and vibrations [30,37,43,45,51,52], disregard for the driver’s normal movements during driving [44], testing on a small non-diverse demographic [28,39,41,43,44,47,51], and insufficient validation of physiological data quality against gold standards [28,30,38,41,42].

**Table 1:** Key developments in in-vehicle biosensing systems: a review by sensor placement.

Sensor Location	Article	Modality	Methodology and Results	Limitations
Steering Wheel	K. Futatsuyama et al. (2014) [37]	ECG PPG	A cuffless blood pressure monitoring system was developed based on the correlation between blood pressure and pulse transit time measured from ECG and PPG signals. The system was trained on 1008 participants and tested on a static steering wheel on 92 participants. It attained a mean error of −1.30 mmHg with a standard deviation of 5.95 mmHg.	The system relies on contact ECG and PPG measurements through the steering wheel. The system was only tested on a static steering wheel without considering road noise, vibrations, and driving conditions.
	B.G. Lee., et al. (2016) [38]	PPG	HRV and respiratory rate derived from ECG obtained through a conductive fabric on the steering wheel were used in a kernel fuzzy c-means wavelet method to identify driver’s fatigue. The system was tested on 12 participants under normal driving conditions in the city. It achieved the best performance of 96% true and 8% false detection rate when combining all parameters.	The ECG could only be acquired when both hands are on the steering wheel. The quality of the ECG signal and the accuracy and reliability of the derived heart rate were not studied.
	Y.-J Choi et al. (2018) [39]	ECG	ECG was derived from a steering wheel covered with a conductive fabric-based dry electrode material, manufactured by an electroplating method. The derived ECG was compared to the clinical-grade ECG during driving. The SNRs of the measured ECG signals were 15.11 dB in the idling state and 12.69 dB in the driving state	The system was tested on only one participant. The accuracy and reliability of ECG-derived heart rate were not studied. ECG could only be acquired when both hands were on the steering wheel.
	J.K. Park (2019) [40]	Radar	A radio frequency sensor based on double voltage-controlled oscillators (VCOs) combined with a switchable phase-locked loop (PLL) was designed and mounted on the steering wheel to measure HR and RR. The method showed a strong correlation with reference measurements: 0.98 for men’s respiration rate, 0.92 for men’s heart rate, 0.88 for women’s respiration rate, and 0.88 for women’s heart rate.	The system was tested on only two individuals. The experiments were performed only in a static-vehicle environment.
	B. Babusiak et al. (2021) [53]	ECG PPG IMU	Integrated electrocardiograph, oximeter, and inertial unit in the steering wheel for recording and analyzing driver’s movement patterns. The system was evaluated under laboratory and real-life conditions. The quality of ECG and PPG signals was compared subjectively with a BIOPAC device.	The experiments were solely conducted on one individual. The precision of heart rate and other vital signs was not studied.
	J.M. Warnecke, et al. (2022) [54]	ECG	Flexible and thin electrodes made of polyurethane were designed for long-term ECG. The system was tested on 19 participants in different driving scenarios. Developed electrodes provided reliable recordings covering about 45.62% of driving time.	The ECG could only be acquired when both hands are on the steering wheel.
Car seat	R. Fu & H. Wang (2014) [43]	ECG EMG	Features extracted from capacitive ECG and EMG derived through electrodes mounted in the car seat were used to determine the driver’s fatigue. An accuracy of 0.87% was achieved.	The study was performed on only 8 subjects in a static driving simulator. The effects of road noise and vibrations were not considered.
	E. Schires, et al. (2018) [44]	Radar	A UWB pulsed radar was mounted in the seat backrest to measure instantaneous heart rate and breathing rate when the car is moving. Compared to a BioHarness, HR estimation achieved errors as low as 1.82% in static measurements and 13% in dynamic measurements.	The system was tested on only three individuals The system was tested on the passenger seat.
	L. Leicht, et al. (2018) [45]	ECG	Quality of capacitive ECG was compared against conventional ECG on 10 patients after a major cardiac event on a driving simulator. Cardiologists found cECG quality lower than reference ECG, often with algned assessments or minor grade differences.	The driving simulator was static, and the effects of road noise and vibrations were not considered. The effect of thick outerwear was not studied.
	D.U. Uguz, et al. (2020) [46]	ECG	Capacitive ECG (cECG) electrodes were designed for monitoring patients with implanted cardiac pacemakers. Conducted with 20 pacemaker patients, the method demonstrated cECG’s capability to detect pacemaker spikes, with F1-scores ranging from 0.62 to 0.88.	The study was focused on patients with pacemakers The quality of ECG and the accuracy of HR estimates were not studied.
	C. Loss, et al. (2021) [49]	Radar	A textile-based bio-radar system was proposed for non-invasive monitoring of respiration. It was observed that the textile antennas have the same capability to detect respiratory signals when compared with a conventional substrate antenna	The system was tested on only 1 participant The participant was instructed to remain still, without engaging in any driving simulations.
Seat belt	H. J. Baek, et al.(2009) [47]	ECG PPG EDA Piezoelectric	Different biosignals were measured in a ubiquitous car using non-intrusively installed sensors on the seat belt, steering wheel, and car seat to estimate driver’s stress during driving. The correlation coefficients with reference biosignals from a Biopac system were 0.986 for ECG, 0.970 for PPG, and 0.965 for respiration.	The system was tested on only four individuals. The system relied on proper contact of the driver to the sensors.
	X. Ji, et al.(2022) [48]	ECG Piezoelectric	An airline point-of-care system containing hybrid ECG, breathing, and motion signals was proposed. The system, tested on 18 adults for detecting sleep apnea–hypopnea syndrome, achieved an accuracy rate of up to 85%.	The sensor was not tested in real-life settings. The performance of heart rate and respiration rate predictions was not analyzed.
Dashboard and Instrument Panel	Q. I. Zhang, et al. (2018) [51]	Cam	An automatic facial tracking algorithm was used to transform facial images obtained from a NIR camera into time-series signals and estimate heart rate. Tested on 20 drivers, the system achieved an accuracy of 95% compared to a commercial finger PPG sensor.	The system did not provide instantaneous heart rate The HR measurement results during driving were only reported for five participants.
	F. Wang, et al. (2022) [50]	Radar	A millimeter-wave radar was placed on the windshield and under the steering wheel to measure heart rate and respiration rate in real-life settings. Achieved median errors of 0.16 respiration per minute and 0.82 beats per minute in estimations.	The system was tested on only four individuals.

Future research should focus on developing accurate and comfortable sensors without direct contact, advancing contactless technologies like radar and cameras, and addressing the current limitations. This includes handling environmental variables, considering the driver’s natural movements, broader demographic testing, and rigorous validation of data quality. These improvements could enhance the feasibility and reliability of in-vehicle biosensing, leading to safer health-aware driving experiences.

**Figure 3:**
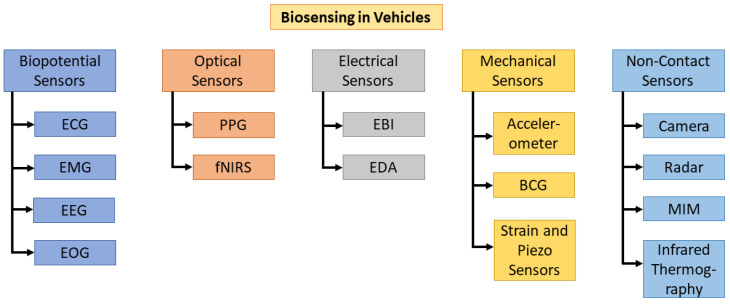
Distribution of sensors across different categories.

The remainder of this section explores various sensing modalities suitable for in-vehicle biosensing and their potential applications. A detailed breakdown of the sensor categories and their types is provided in Figure 3.

### 2.1. Biopotential Sensors

Biopotentials are generated by excitable cells in the body’s nervous, muscular, glandular, and cardiovascular systems. These electrical signals can be measured on the skin’s surface using electrodes. Various biopotential sensors have potential for use in vehicle biosensing.

#### 2.1.1. Electrocardiogram (ECG)

ECG is crucial for in-vehicle biosensing due to its accuracy in capturing heart activity, which is essential for monitoring heart rate (HR) and heart rate variability (HRV) [26,55,56]. Effective ECG monitoring can be achieved using one or two electrodes [33,57,58]. Different types of electrodes have been adapted for in-vehicle use, including gel electrodes that offer high signal quality but may cause discomfort [59], dry electrodes that are more convenient but can have increased impedance [60,61], and textile electrodes that are integrated into the seat or steering wheel, allowing seamless monitoring without direct skin contact [39]. Additionally, capacitive electrodes can acquire ECG signals through clothing, reducing intrusion [32,43,45,46,62].

Signal conditioning circuits are essential for enhancing low-amplitude biopotentials like ECG [63]. However, motion artifacts, which are common during driving, can affect accuracy, especially for HRV estimation—a critical indicator of drowsiness [64]. Adaptive filtering techniques can mitigate these artifacts, ensuring stable and accurate monitoring [65].

#### 2.1.2. Electromyography (EMG)

EMG assesses muscle activity and is valuable for tracking driver behavior and fatigue. Skin EMG captures electrical potentials from muscle contractions and helps to detect driver fatigue. While traditional EMG sensors are cost-effective, they can distract drivers due to connecting wires. Wireless dry electrodes, affixed with elastic armbands, offer greater mobility and reduce motion artifacts [60,66,67].

EMG also monitors stress and workload, as shown in studies on driver stress during vehicle platooning and physical strain during navigational maneuvers [68,69].

### 2.2. Optical Sensors

Optical sensors detect physiological signals by analyzing how light interacts with biological tissues. They are widely used for non-contact or minimally invasive monitoring due to their affordability, ease of integration, and ability to capture vital signs like heart rate, blood oxygenation, and respiration. These sensors typically rely on measuring either light absorption or reflection to derive physiological parameters and are particularly suitable for wearable and in-vehicle applications where unobtrusiveness is critical.

#### 2.2.1. Photoplethysmography (PPG)

PPG monitors blood volume changes and heart rate by measuring light absorption variations using an LED and a photodetector. Traditionally, red and infrared (IR) light were used, but green light has become common, especially in smartwatches, due to its better perfusion information for accurate heart rate monitoring. Red light and near-infrared (NIR) light penetrate deeper but are less optimal for heart rate monitoring [70].

PPG technology can also extract heart rate variability (HRV), pulse transit time (PTT), oxygen saturation (SpO2), and other cardiovascular data. Combining PPG with ECG provides a comprehensive view of the cardiovascular system, useful for monitoring driver stress through parameters like PTT and HRV [33,71].

#### 2.2.2. Pulse Oximetry

Pulse oximetry is an optical technique used to estimate blood oxygen saturation (SpO2) by analyzing the differential absorption of red and infrared light through perfused tissue [72]. While closely related to PPG, pulse oximetry focuses specifically on oxygen saturation by comparing the pulsatile (arterial) absorption at different wavelengths. In driving contexts, maintaining adequate SpO2 levels can be essential for detecting hypoxia, sleep apnea, or respiratory distress, which may compromise driving performance. This method can be integrated into ear clips, fingertip sensors, or even smartwatches for continuous monitoring.

### 2.3. Electrical Sensors

Electrical sensors in vehicles gauge changes in electrical properties such as impedance, inductance, or capacitance using external voltage or current.

#### 2.3.1. Electrodermal Activity (EDA)

Electrodermal activity (EDA), or galvanic skin response (GSR), reflects autonomic nervous system responses through skin conductivity changes, providing insights into emotional and physiological states. EDA can aid in detecting driver stress and distraction.

Implementing EDA in vehicles is challenging due to its contact-based nature, requiring electrodes attached to the skin. The measurement location also affects the signal; traditional hand-based EDA is impractical in cars, although steering-wheel-based sensors are being explored.

Despite these challenges, EDA has shown potential for driver monitoring. Chen et al. used EDA with ECG and respiration for stress detection [73]. Amidei et al. developed an embedded system to process EDA and PPG signals in real time, accurately detecting heart rate (HR) and skin conductance response (SCR) peaks [29]. EDA has also been effective in detecting distraction levels and differentiating among drivers with diabetes [74]. Additionally, combining EDA with eye tracking and ECG has assessed the impact of vehicle settings on driver stress [75].

#### 2.3.2. Electrical Bioimpedance (EBI)

Electrical bioimpedance (EBI) measures the impedance of biological tissues using a small applied current, offering insights into body composition, hydration, and cardiac and respiratory activity [76]. EBI can be integrated into steering wheels, seats, or seatbelts for continuous unobtrusive health monitoring. For example, seat-embedded sensors can detect impedance changes related to heart and respiration rates, helping to detect stress, fatigue, or medical emergencies.

However, traditional EBI requires multiple direct-contact electrodes, limiting its practicality in vehicles. While capacitive coupling could reduce contact, it becomes less effective at frequencies over 200 kHz, which are needed for accurate data. Advancements in multi-electrode patches or textile-based electrodes integrated into car interiors could address these challenges, making EBI more feasible for unobtrusive monitoring in cars. Further research is needed to optimize bioimpedance measurement techniques for this purpose.

In addition, advanced artifact detection and removal techniques are required that can deal with car motion and driver movements [77].

### 2.4. Mechanical Sensors

Mechanical sensors monitor driver activities and physiological states in vehicles, focusing on pressure, body deformation, temperature, and acceleration.

#### 2.4.1. Accelerometers

Accelerometers measure vehicle acceleration, vibrations, and impacts, crucial for enhancing ride comfort, monitoring driver behavior, and supporting safety-critical functions in advanced driver assistance systems (ADAS) like collision avoidance and automatic emergency braking. They can also monitor respiration and heart rate by capturing vibrations caused by heartbeats and respiratory movements when placed near the chest, producing seismocardiogram (SCG) or ballistocardiogram (BCG) signals [78,79,80].

Additionally, accelerometers serve as independent measures of noise and vibration within adaptive noise cancellation (ANC) frameworks [81]. Recent research integrates accelerometers with radar and ECG technologies to distinguish physiological signals from vehicle-induced noise, achieving over 98% accuracy in heart rate measurements under real driving conditions [62,82].

#### 2.4.2. Strain and Piezoelectric Sensors

Strain and piezoelectric sensors convert mechanical energy from physical stress and movements into electrical signals, providing insights into driver posture and body movements [83]. These seat-mounted sensors can monitor pressure exerted by the driver, indicating fatigue or discomfort, and detect subtle changes in seating position, signaling drowsiness or distraction [84].

Additionally, they can be used for respiration monitoring, offering insights into stress and fatigue levels [85]. Other methods for respiration monitoring, such as capacitive sensors and acoustic sensors, have also been explored for detecting breathing patterns [86,87].

#### 2.4.3. Ballistocardiography (BCG)

BCG, an early medical sensing method, offers the potential for non-contact monitoring by integrating it into vehicle seats. BCG detects the mechanical activity of the heart and respiration through pressure- or strain-gauge-based sensors, capturing movements caused by the heart, lungs, or blood pulse [28,33]. Unlike ECG, it monitors mechanical movements, making it a simpler and cost-effective option.

BCG accuracy is affected by variability in body positioning [33]. Early efforts, such as using the quasi-piezoelectric ferroelectric EMFiTM mat in the SMART car’s passenger seat, showed potential but faced challenges like engine vibrations that reduced data consistency in moving vehicles [88].

A 2018 study by Wusk and Gabler improved BCG monitoring by using a fluid-filled bladder and pressure transducer in a Ford Mustang’s passenger seat. This system accurately measured heart and respiratory rates under controlled conditions, marking a significant advancement in in-vehicle BCG monitoring [89]. However, challenges with vibration and movement during driving still need to be addressed.

### 2.5. Non-Contact Sensors

Non-contact sensors are crucial for assessing a driver’s physiological state without physical contact, enhancing safety and performance. These include cameras and radar technology, which capture physiological signals to monitor vital signs and driver attentiveness.

#### 2.5.1. Cameras

Cameras, spanning different frequency bands, are essential for non-contact physiological monitoring in vehicles. These include visible (RGB or monochrome) cameras for facial recognition and behavior analysis; near-infrared (NIR) cameras for tracking heart rate and facial movements in low-light conditions; and long-wave infrared (LWIR) or infrared thermography (IRT) for detecting heat signatures and physiological changes like temperature.

Visible and NIR cameras are frequently employed for capturing facial features and physiological signals, such as heart rate, respiration, and blink rate, without physical contact. Camera-based photoplethysmography (cbPPG) allows real-time cardiovascular monitoring by analyzing skin tone changes to assess driver health and alertness, even under varying lighting conditions [90,91,92,93]. Recent advancements in cbPPG have shown up to 95% accuracy in heart rate estimation using facial tracking algorithms [51,94]. The integration of cbPPG with advanced machine learning is a promising avenue for enhancing precision and reliability, particularly for continuous real-time health assessment in dynamic environments.

IRT excels in capturing heat signatures, making it effective for monitoring physiological changes related to stress, drowsiness, or temperature fluctuations. It has been shown to capture facial features like eye closure and head pose with up to 90% success in identifying drowsiness [95,96]. However, differentiating between drowsiness and other factors, like high ambient temperatures, remains a challenge [97].

Machine learning techniques significantly boost the performance of camera-based monitoring across all frequency bands [98]. Algorithms such as Long Short-Term Memory (LSTM) networks have been applied for drowsiness detection using IR data [99]. Self-supervised learning with masked image modeling has achieved up to 99.6% accuracy in identifying driver distraction [100]. Moreover, lightweight vision transformers combined with convolutional neural networks (CNNs) have demonstrated over 80% detection accuracy for various distraction categories in RGB datasets [101]. Real-time deep CNNs deployed on embedded systems have achieved 97.5% accuracy in distraction detection across multiple datasets [102], while CLIP-based vision–language models have set new benchmarks in detecting distracted driving with state-of-the-art performance [103].

Despite the advancements, challenges remain in ensuring robustness across different lighting conditions, head movements, and facial obstructions. Privacy concerns also arise with continuous monitoring, necessitating secure data handling and proper user consent. The integration of infrared illumination and more sophisticated algorithms can help to maintain accuracy and reliability in challenging environments. Future research should aim to combine cbPPG, IRT, and NIR modalities with advanced machine learning models to build a more comprehensive, robust framework for monitoring driver health and alertness. This multi-spectral approach could provide richer physiological insights, improving the precision of driver state detection and enhancing vehicle safety [96,104].

#### 2.5.2. Radar

Radar technology provides a non-intrusive way to monitor vital signs like heart rate (HR) and respiration rate (RR) without direct contact [105,106]. Using continuous-wave and pulse radars, these systems detect body motions linked to physiological activities, enabling accurate heart rate (HR) and respiration rate (RR) estimations.

A pioneering study employed a millimeter-wave radar (mmWave) system to estimate respiration rate (RR), heart rate (HR), and interbeat intervals (IBIs) with high precision, even in the presence of motion artifacts [50]. The system’s motion compensation module and periodicity check effectively isolated vital signals, yielding median errors of 0.16 respirations per minute (RPMs) for RR, 0.82 beats per minute (BPMs) for HR, and 46 ms for IBIs, highlighting its vehicle safety potential. Another study introduced a radio frequency vital sign sensor with double voltage-controlled oscillators (VCOs) and a switchable phase-locked loop (PLL) to remotely detect lung and heart movements in a static-vehicle environment, demonstrating its real-time monitoring capability [40].

Radar technology’s capacity to monitor vital signs through clothing or walls has also been investigated [44]. With body-coupled antennas and ultra-wideband (UWB) pulsed radar, this method addresses front monitoring limitations. By combining phase-based detection with frequency estimation, the system achieves robust HR and RR measurements, indicating its potential for integration into smart car seats for driver monitoring. Recent research combined spatial–temporal-circulated gray-level co-occurrence matrix (STC-GLCM) features with cardiopulmonary physiological data for accurate people counting, motion recognition, and presence detection using IR-UWB [107]. The method achieved over 97% classification accuracy in both stationary and moving vehicles.

Future research should focus on improving the accuracy and reliability of non-intrusive vital sign monitoring in diverse driving conditions. Integrating these technologies with AI and machine learning could provide predictive insights into driver health, enabling more personalized ADAS functionalities. The key challenges include potential interference from electronic devices, high processing power demands, data security, and ensuring consistent performance in dynamic environments.

### 2.6. Emerging Biosensing Technologies

This section explores emerging biosensing technologies with potential for future integration into in-vehicle systems.

#### 2.6.1. Electroencephalography (EEG)

EEG monitors brain activity and cognitive functions, making it valuable for brain–computer interfaces (BCIs) and brain-controlled vehicles (BCVs) [23,108]. EEG data can translate into vehicle commands, bypassing traditional interfaces, with significant correlations shown between fatigue, drowsiness, and EEG frequency bands such as delta (1–3 Hz), theta (4–7 Hz), and alpha (8–12 Hz) [23,109,110].

In-vehicle EEG faces challenges like motion artifacts and user discomfort with headbands. However, advancements such as the Muse headband with soft materials and dry electrodes and the OpenBCI Ultracortex, featuring reduced-pressure designs, offer promising solutions [111,112]. Additionally, integrable sensors in headrests or ceilings enhance comfort and usability [113]. These, combined with improvements in signal processing, miniaturization, battery life, and wireless communication, pave the way for more effective in-vehicle EEG applications.

#### 2.6.2. Electrooculography (EOG)

EOG measures eye movements by capturing corneo-retinal potential differences, offering reliable data for monitoring driver fatigue and drowsiness [114,115,116,117]. EOG is less prone to noise compared to EEG. A study using unsupervised machine learning demonstrated that a convolutional neural network could automatically extract EOG features, outperforming traditional methods [115]. Another study linked eye and eyelid movements with pilot fatigue, confirming that fatigue-induced performance declines are measurable and physiologically correlated [117].

#### 2.6.3. Magnetic Impedance Monitoring (MIM)

MIM offers a non-invasive approach to monitoring cardiovascular health in vehicles by using a magnetic field to induce electrical currents within the body’s tissues [118]. This technique has been used to monitor heart rate, lung activity, and blood volume and flow changes with each heartbeat [28,118]. While integrating MIM into automotive environments presents challenges, such as system complexity and unobtrusive sensor placement, its potential for real-time cardiovascular data collection enhances driver safety.

#### 2.6.4. Functional Near-Infrared Spectroscopy (fNIRS)

fNIRS emits near-infrared light into the scalp, where absorption varies with blood oxygenation, allowing it to measure brain activity [119,120]. This technology provides valuable insights into cognitive processes, making it useful for monitoring driver attention, workload, and stress levels [121,122].

Recent studies using fNIRS in vehicles revealed heightened brain activity during driving risks, with increased awareness in complex traffic and unexpected hazards [120,123]. A voice-only driver assistant system was found to cause higher cognitive workload compared to visual agents [122]. Advances in portable systems, such as Brite24 and NIRSport2, enhance fNIRS usability in vehicles [124].

Future research should focus on integrating fNIRS with other biosensing technologies, enabling real-time driver monitoring and feedback to improve safety and vehicle system design.

#### 2.6.5. Chemical Sensors

Chemical sensors can play an important role in monitoring the in-cabin environment and potentially the driver’s health [125,126]. Gas sensors can detect the presence of hazardous gases like carbon monoxide (CO), nitrogen oxide (NOx), and volatile organic compounds (VOCs) emanating from vehicle systems or the external environment, ensuring acceptable air quality and alerting occupants to potential risks. Emerging research also explores the use of breath analysis sensors to non-invasively detect biomarkers associated with fatigue, stress, or even certain medical conditions [127]. These sensors could analyze exhaled breath for specific chemical compounds. Challenges include achieving high sensitivity and selectivity in complex cabin environments with varying humidity and temperature, as well as ensuring long-term stability and low power consumption for automotive integration.

#### 2.6.6. Acoustic Sensors

Acoustic sensors, primarily microphones, offer a non-contact method for gathering information about the driver and the vehicle environment [128]. Analyzing speech patterns can reveal signs of drowsiness (e.g., slurred speech and long pauses), stress (e.g., changes in tone and rate), or cognitive load [129]. Beyond speech, acoustic analysis can detect physiological sounds like coughing or sneezing, which could be relevant for health monitoring. Furthermore, monitoring the acoustic environment within the cabin can provide insights into driver distraction due to conversations or external noise. Advanced signal processing and machine learning algorithms are crucial for extracting meaningful information from noisy in-vehicle audio. The challenges include privacy concerns related to continuous audio recording and the need for robust noise cancellation and speaker diarization techniques.

#### 2.6.7. Blood Pressure Sensors

Continuous and non-invasive blood pressure monitoring in vehicles presents a significant challenge but holds substantial potential for driver health safety. Traditional cuff-based methods are impractical during driving [130]. Research is exploring cuffless blood pressure estimation techniques using various sensor modalities. Photoplethysmography (PPG), already mentioned, can be used to derive blood pressure trends, although absolute accuracy remains a challenge [131]. Other emerging approaches include using radar- or ultrasound-based sensors to measure subtle changes in arterial wall movement or blood flow [132]. Integrating reliable and unobtrusive blood pressure monitoring could enable early detection of critical cardiovascular events or chronic conditions affecting driving safety. The challenges lie in achieving clinical-grade accuracy with non-contact or minimally intrusive methods and addressing potential motion artifacts within the vehicle.

## 3. Vehicle-Based Biosensing Applications

**Figure 4:**
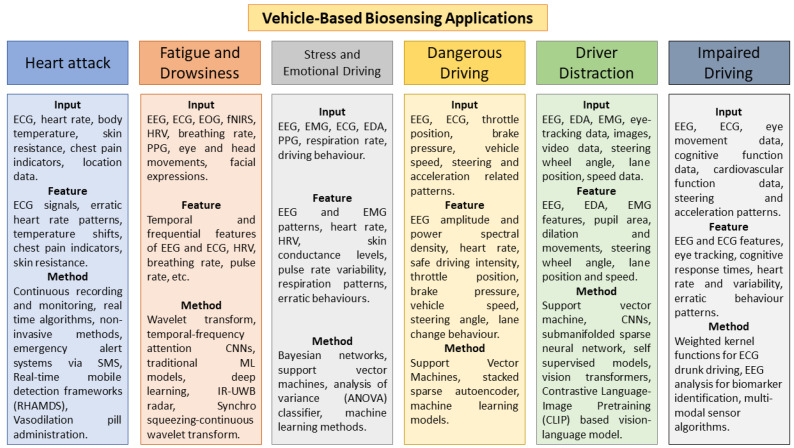
Overview of vehicle-based biosensing applications.

This section explores the diverse applications of biosensors for in-vehicle health monitoring, as summarized in Table 2. Figure 4 visually depicts these advancements. Biosensing technologies hold promise for detecting critical events like heart attacks, fatigue, stress, dangerous maneuvers, and impaired driving.

When evaluating these biosensing systems, it is crucial to consider specific performance metrics. While overall accuracy—the total proportion of correct predictions—provides a general indication of performance, it can be misleading, particularly in scenarios where the conditions being detected (e.g., a heart attack or extreme fatigue) are rare. Therefore, more nuanced metrics are vital: sensitivity (or recall) measures the system’s ability to correctly identify actual positive instances (e.g., truly detecting a heart attack when it occurs); specificity quantifies its capacity to correctly identify actual negative instances (e.g., confirming a driver is not fatigued when they are alert); and precision indicates the proportion of positive predictions that were genuinely correct. The F1-score, as the harmonic mean of precision and sensitivity, offers a balanced single metric, which is particularly valuable for assessing performance in applications with imbalanced class distributions, providing a more robust indicator of a system’s overall effectiveness. Given the extensive number of references reviewed in this study, we primarily focus on overall accuracy as a global performance metric, acknowledging its limitations but recognizing its utility for broad comparison across a wide range of systems.

The following sections will delve deeper into each of these vital applications, highlighting their current capabilities and future potential.

### 3.1. Detection of Heart Attack

Heart disease significantly increases the risk of vehicular accidents, especially through heart attacks, which pose a major concern on the road [57]. Early detection of heart attack symptoms, such as erratic heart rate, temperature shifts, and chest pain, is crucial for reducing these risks [140]. Wearable devices monitoring these vital signs offer a solution by alerting drivers and health authorities early, potentially preventing accidents and saving lives. Ensuring accurate symptom detection without false alarms is vital, highlighting the need for user-friendly designs providing clear, actionable alerts.

In [133], a low-cost, low-power-consumption wearable ECG device was developed for driver heart attack detection, issuing immediate alarms upon detection. In [57], a single-lead ECG system embedded in a steering wheel monitored driver heart health, employing a two-stage machine learning model with promising accuracy. In [141], an interactive steering wheel system continuously monitored vital signs to detect heart attacks in drivers with a history of heart disease, guiding them to safety upon detection. In [142], a real-time heart attack mobile detection service (RHAMDS) modeled smart cars as mobile nodes to prevent accidents caused by heart attacks and improve emergency response.

Advancing wearable technology for heart attack detection entails minimizing false positives and integrating with emergency services to enhance predictive capabilities, enabling more effective early detection systems.

### 3.2. Detection of Fatigue and Drowsiness

Fatigue and drowsiness significantly endanger driving safety, often due to overexertion, monotony, or sleep deprivation [143]. Techniques to detect these states utilize physiological signals, such as EEG, ECG, EOG, and fNIRS, alongside observations of eye and head movements to assess alertness levels [119,144,145].

Recent studies have introduced innovative methods for detecting driver fatigue and drowsiness [110]. Utilizing EEG signals and a Flexible-Analytic-Wavelet-Transform-based machine learning method, a recent study effectively detected driver fatigue with accuracies of 97.10% for fatigue states and 97.90% for rest states, demonstrating real-world potential [146]. Similarly, single-channel EEG signals were analyzed using a novel temporal–frequential attentional convolutional neural network (TFAC-Net) for drowsiness detection, achieving an F1-score of 76% [23]. In a different study, recurrence plots were combined with a CNN model to analyze heart rate variability features, achieving a 70% accuracy rate in detecting drowsiness [27]. Another study introduced an elastic dry electrode for real-time ECG signal acquisition from the palm, simplifying detection and reducing skin infection risks. Employing a periodic man–machine interaction mode (PMIM) to assess driver fatigue, it demonstrated effective fatigue mitigation, with recognition accuracies ranging between 94% and 99% across various experiments [134]. A hybrid model combining deep learning and traditional methods for heart rate variability (HRV) features achieved high accuracy (around 98%) in simulations [58].

Hybrid methods combining different sensing modalities have also been explored. For example, a study combined EEG and ECG signals and achieved up to 95.4% accuracy in classifying drowsiness levels [135]. Future research should focus on improving robustness and accuracy under dynamic driving conditions, developing algorithms to filter motion artifacts and integrate data from multiple sensors, and incorporating vehicular indicators into machine learning algorithms for deeper insights into driver condition [147].

### 3.3. Detection of Stress and Emotional Driving

High levels of stress or negative emotional states can significantly impair a driver’s focus and reaction times, leading to risky driving behaviors [148].

Monitoring the driver’s physiological signals offers insights into their stress and emotional status. Technologies such as EEG for brain activity, EMG for muscle tension, and ECG for heart activity, alongside EDA and PPG measurements, serve as valuable indicators [68,136]. For example, a real-time stress detection method using physiological signals (ECG, EDA, and respiration) and driving behavior achieved a 96% accuracy rate [136]. Utilizing EEG signals, a study applied a support vector machine (SVM) to detect driver stress with a 97.95% accuracy rate [137]. Another study identified ECG as the most effective signal for measuring driver stress, achieving an accuracy of 75.02% [26]. In [149], hand and foot GSR was used alongside an analysis of variance (ANOVA) classifier to classify stress levels in drivers with an accuracy of 95.83%.

Advanced driver assistance systems (ADASs) could leverage physiological signals to detect drivers’ stress or negative emotional states and activate interventions such as playing calming music or issuing safety reminders, contributing to safer driving practices.

### 3.4. Detection of Dangerous Driving

Dangerous driving poses risks to drivers and other road users, often characterized by erratic lane changing and acceleration patterns [66].

A study by Yan et al. utilized EEG and driving data to classify driving styles, achieving an SVM classification accuracy of about 80% [138]. They found distinct power spectral density (PSD) patterns in different brain regions associated with conservative and aggressive driving styles, highlighting the potential of EEG patterns in identifying driving styles. In another study by Li et al., a stacked sparse autoencoder model was trained on vehicle-related data, obtaining micro-recall and micro-precision of 92.66% and 92.69%, respectively [150]. This model effectively learned generic driving behavior features from throttle position, brake pressure, vehicle speed, and steering-related data. In another study, Safe Driving Intensity (SDI) and Cardiac Reaction Time (CRT) were introduced as novel metrics using ECG data to assess correlations between driver heart activity and vehicle movement [151]. These metrics proved highly effective in identifying stressful driving scenarios.

Continuous monitoring of the driver’s ECG can help to control risks as mental workload increases heart rate during lane changes [152]. Koh et al. introduced Safe Driving Intensity (SDI) and Cardiac Reaction Time (CRT) as novel features, derived from ECG data, which were highly effective in detecting stressful driving situations.

The integration of diverse sensors and machine learning technologies in vehicles holds promise for enhancing dangerous driving detection. This approach leverages acceleration and deceleration tracking, auditory alert systems, and advanced physiological sensing to provide comprehensive insights into driver behavior.

### 3.5. Detection of Driver Distraction

Driver distraction poses significant risks on the road, contributing to numerous accidents [3]. Distractions are categorized into cognitive, visual, and manual types, each requiring specific monitoring methods [153].

EEG and EDA modalities effectively identify cognitive distractions [17,154,155]. Visual distractions can be monitored using frontal cameras or EOG sensors [156], while manual distractions can be tracked using EMG sensors and accelerometers.

In a study by Smith et al., EEG revealed distinct responses in the frontal cortex to cognitive distractions, impairing driving performance [154]. Another study employed a wristband to measure EDA, achieving a cross-validation accuracy of 94.81% in identifying distractions [17].

Various features, from eye-tracking and physiological data, along with vehicle kinematics, have been investigated to identify distracted drivers, achieving an average accuracy of 90% [157].

Recent advancements include the use of deep learning models like deep convolutional neural networks and neuromorphic event cameras for real-time distraction detection [102,158]. Self-supervised learning and vision–language models have also shown promise in accurately detecting distractions [100,103].

Future research may focus on refining sensor fusion techniques and developing robust algorithms to detect and mitigate distractions effectively. Additionally, exploring innovative sensor technologies could further improve detection accuracy and reliability.

### 3.6. Detection of Impaired Driving

Impaired driving, fueled by alcohol, drugs, or medication, poses a grave threat to road safety [159]. While traditional methods rely on post-incident testing, emerging technologies target real-time detection to bolster preventive measures. Sensor advancements and machine learning algorithms offer promising avenues for identifying signs of impairment, such as erratic steering and delayed reaction times. Integrating physiological sensors to monitor eye movement, cognitive function, and motor control aids in assessing impairment levels [139,160].

Wu et al. developed an ECG-based Drunk Driving Detection (DDD) system, achieving an 87.52% accuracy using weighted kernel functions [139]. Recent research utilized EEG biomarkers to evaluate the impact of cannabis intoxication on driving performance, revealing correlations between impaired driving and EEG power in specific frequency bands [160].

Future directions in impairment-driving research involve enhancing multimodal sensor technologies, refining AI algorithms, and exploring integration into vehicles or wearables for proactive detection and mitigation of impairment, thus bolstering road safety [161].

## 4. Overview of Key Algorithms

**Figure 5:**
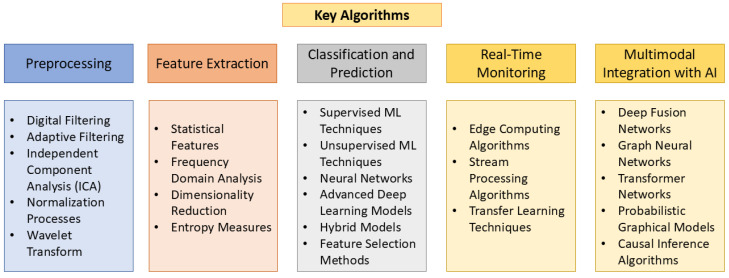
Overview of key algorithms.

The successful integration of biosensing technology in vehicles hinges on powerful algorithms that can accurately process and interpret physiological data. These algorithms, encompassing various machine learning and signal processing techniques, are designed to decipher the complexities of human–vehicle interaction dynamics. Figure 5 illustrates these algorithms, while Table 3 provides a summary. The following sections will delve deeper into their functionalities.

### 4.1. Preprocessing

In vehicle biosensing, preprocessing techniques play a crucial role in enhancing the quality and interpretability of physiological signals. Digital filtering is commonly employed to selectively remove unwanted components from signals, such as power line interference and baseline wander, offering precise frequency characteristics adjustment. Adaptive filtering further complements this by dynamically adjusting its parameters in real time, responding to changes in signal or noise characteristics, which is particularly useful in the variable noise environment of a moving vehicle [167]. Independent Component Analysis (ICA) can be utilized for separating multivariate signals into additive independent components, effectively isolating neural signals from artifacts like eye blinks or muscle movements [168]. Normalization processes, including standardization or min–max scaling, can be used to adjust signal amplitudes to a standard scale, enhancing data consistency across different sessions or subjects. In addition, the wavelet transform can be used to obtain a detailed time–frequency analysis for nonstationary physiological data, allowing for the decomposition of signals into wavelets at different scales to analyze transient features closely and remove unwanted components [110].

### 4.2. Feature Extraction

Feature extraction plays a vital role in the preparation of data for machine learning models, particularly in scenarios where direct application of deep learning may not be feasible or when simplifying raw data is necessary for more effective learning. This process involves distilling essential information from complex physiological signals, enabling the identification of specific patterns or features that are most indicative of the driver’s state, such as stress levels or fatigue. By reducing the dimensionality of the data and highlighting key attributes, feature extraction makes the dataset more manageable and interpretable for traditional machine learning algorithms.

For example, statistical features like mean, standard deviation, and higher-order moments offer straightforward insights into physiological states. Fast Fourier Transform (FFT) and Short-Time Fourier Transform (STFT) enable frequency domain analysis and time–frequency analysis, respectively, critical for understanding heart rate variability and EEG signal changes over time [169]. Principal Component Analysis (PCA) and linear discriminate analysis (LDA) aid in reducing the dimensionality of high-dimensional data, highlighting the most significant features for classification [166,170]. Additionally, entropy measures provide a quantification of signal complexity, which can be indicative of stress levels [171].

### 4.3. Classification and Prediction

In the classification and prediction phase of vehicle biosensing, the focus shifts towards interpreting the extracted features to understand the driver’s physiological state. This step is crucial to identify patterns that indicate stress, fatigue, or other conditions that could affect driving performance.

Machine learning (ML) algorithms, which include supervised and unsupervised methods, play a crucial role in the analysis of physiological signals to predict driver states. Supervised techniques such as support vector machines (SVMs) are widely used for their proficiency in high-dimensional data classification, which makes them adept at differentiating various physiological states [172]. Decision trees and random forest algorithms utilize supervised learning to formulate intuitive classification rules and provide insights into the importance of different characteristics [159]. On the unsupervised spectrum, techniques such as clustering and dimensionality reduction are instrumental in discovering latent patterns within the data, offering valuable insights without relying on prelabeled results [173]. For handling complex or nonlinear relationships in the data, neural networks, including simpler architectures as well as advanced deep learning models such as convolutional neural networks (CNNs) [174] and recurrent neural networks (RNNs) [175], are utilized. These models, which are adaptable for both supervised and unsupervised learning, are adept at capturing intricate patterns within physiological signals. For example, a novel model combining EEG and EOG signals effectively detects driver sleepiness by analyzing alpha wave changes, achieving high accuracy (95% F1-score and 98% mean accuracy) with the aid of LSTM networks and dataset augmentation via generative adversarial networks (GANs) [176]. A similar study introduced a novel methodology to detect multi-level driver fatigue by analyzing ECG, EEG, EMG, and respiratory signals [163]. This approach used a combination of GANs and CNNs, achieving accuracies ranging from 89% to 96%. Recent advances in driver emotion recognition have shifted toward hybrid models that take advantage of the strengths of CNN and RNN, enhancing the accuracy and robustness of detecting complex emotional states in drivers [165]. Transformer, a type of attention-based network, has recently been employed for the detection of driver distraction, using multimodal data to achieve comprehensive and accurate assessments of driver focus and awareness [164].

The primary advantage of deep learning models lies in their capacity to learn directly from raw data, thereby eliminating the need for manual feature extraction by experts, provided that sufficient data is available. These ML strategies collectively contribute to the development of sophisticated and adaptive vehicle biosensing systems capable of accurately predicting driver states.

### 4.4. Real-Time Monitoring

For real-time monitoring in vehicle biosensing, edge computing algorithms stand out by processing data directly on local devices, drastically cutting down response times essential for immediate actions [177]. Real-time decision trees and random forests offer fast and efficient classification, making them well-suited for instant decision-making based on physiological signals. For example, a study focused on predicting drivers’ takeover performance in conditionally automated driving by analyzing physiological and environmental data before a takeover request [162]. Using data from human-in-the-loop experiments, where drivers engaged in non-driving tasks were asked to take over control, the study utilized heart rate, EDA, eye-tracking metrics, and driving conditions. The random forest classifier emerged as the most effective method, achieving 84.3% accuracy and 64.0% F1-score in predicting takeover performance, recommending a 3-s prediction window.

Stream processing algorithms, crucial for managing continuous data streams, ensure dynamic and ongoing monitoring of driver states. Adaptive filtering dynamically adjusts to changing signal conditions, preserving data quality in the fluctuating environment of a vehicle. Additionally, transfer learning techniques allow quick adaptation of pre-trained models to new tasks with minimal data, speeding up the deployment of real-time monitoring solutions [178].

### 4.5. Multimodal Sensing and Contextual Data Integration with AI

Integrating multimodal sensing and contextual data with AI offers a powerful approach for in-vehicle physiological monitoring [179]. Combining data from EEG, ECG, facial cues, body acceleration, and contextual information (e.g., vehicle dynamics, environmental conditions, and time of day) can help to overcome the limitations of individual sensors and enhance the detection of fatigue, distraction, and stress-related impairments. Techniques like Kalman filtering can fuse noisy signals from various sensors (e.g., heart rate monitors, respiratory sensors, and skin conductance sensors) to provide a more robust and accurate assessment of a driver’s physiological state over time [180]. Deep fusion networks can learn complex relationships between these physiological signals and contextual information such as driving behavior, environmental conditions, and driver profiles to detect stress, fatigue, or other critical states [181]. Furthermore, graph neural networks can model interdependencies between physiological signals and contextual factors, enabling a more holistic understanding of the driver’s condition [182]. These advanced AI algorithms pave the way for proactive safety systems that can adapt to the driver’s real-time physiological needs, potentially preventing accidents and enhancing overall driving safety.

For example, Cao et al. (2025) developed a multimodal neural network approach that integrates EEG, ECG, and facial image data using a feature-coupling mechanism [183]. Their system achieved an accuracy of 98.41% in detecting driver fatigue using the DROZY dataset, highlighting the potential of dynamic feature interaction in improving classification performance. The DROZY dataset is a publicly available multimodal database collected from subjects experiencing real drowsiness, containing synchronized physiological signals (like EEG, EOG, ECG, and EMG) and near-infrared video data to aid in developing and evaluating drowsiness monitoring systems.

AI plays a critical role in this integration. It can fuse data from diverse sources, identify complex patterns, and adapt to individual driver baselines. Craye et al. (2016) proposed a multimodule system combining visual, auditory, and physiological data through hidden Markov models and Bayesian networks, achieving 98.4% accuracy for fatigue and 90.5% for distraction detection [184]. Their architecture allows independent module operation and contextual inference, a strategy that supports flexible real-time monitoring

Sensor fusion is also essential for reliable physiological monitoring in motion-heavy environments. Warnecke et al. (2021) demonstrated that combining ECG, PPG, BCG, and image-based PPG using CNNs improved heartbeat detection accuracy up to 97.24% at rest and 94.38% during driving, showcasing how sensor fusion enhances robustness in dynamic vehicle settings [185].

In practical applications, integrating biological data with behavioral observations offers insights beyond traditional sensors. Yoshida et al. (2024) evaluated older drivers’ fatigue using a simulator paired with wearable sensors [186]. While physiological metrics like heart rate variability showed minimal change, arousal levels and reaction times varied noticeably, suggesting that combining multiple indicators is key to effective fatigue assessment in aging populations.

Nemcová et al. (2020) provided a comprehensive review of multimodal fatigue and stress detection systems, highlighting the diversity of signals—biological, vehicular, and visual—used in both research and commercial settings [187]. They stressed the importance of high-quality datasets and real-world validation to improve system reliability in future autonomous or semi-autonomous vehicles.

Despite the promise, challenges in data synchronization, dimensionality reduction, model interpretability, and privacy must be addressed. Emerging technologies like edge computing and federated learning may offer practical pathways for real-time privacy-preserving implementation in future vehicles.

## 5. Industrial Prototypes

Driver status monitoring (DSM) technology, a key innovation from leading automakers, significantly improves road safety through real-time monitoring of a driver’s state [188]. These systems leverage a variety of sensors to analyze aspects like steering patterns and even advanced drowsiness detection. The system can alert the driver when a potentially dangerous situation is identified. In this section, we briefly introduce some of these systems that incorporate physiological sensing into DSM technology. Table 4 showcases the latest commercially available prototypes.

### 5.1. Safety Enhancement Systems

Mercedes-Benz ATTENTION ASSIST: This system mainly uses steering behavior sensors combined with a suite of vehicle motion sensors to detect irregular driving patterns indicative of drowsiness [195]. In addition, it may incorporate camera-based systems to monitor eye blink rates and gaze direction.

Tesla Driver Engagement Monitoring: Tesla employs cabin cameras focused on the driver to analyze head position, eye movement, and blink rate, ensuring that the driver’s attention remains on the road, especially when Autopilot is activated [197].

Toyota’s Steering Wheel with Heart Rate Sensors: This prototype incorporates similar ECG sensors within the steering wheel, providing real-time monitoring of the driver’s heart rate to identify sudden health problems or stress [63].

Ford’s Health-Focused Technologies: Ford has developed health monitoring technologies, including an integrated non-contact ECG for heart health monitoring within the driver’s seat and the capability to display glucose levels on the vehicle’s dashboard for diabetes management [200].

Volvo Intoxication and Distraction System: Volvo has developed a comprehensive DSM system that not only tracks car movements in relation to road markings for drowsiness detection but also includes cameras aimed at the driver to assess for signs of intoxication or distraction. This system is particularly notable for its phased response strategy, which transitions from manual to automated control to ensure safety in critical situations [198].

### 5.2. Comfort and Wellness Optimization

BMW and Wearable Device Interface: BMW’s system employs skin conductance sensors to detect stress levels and incorporates steering wheel sensors that monitor heart rate [201]. This setup enables the vehicle to automatically adjust features like radio volume or block phone calls in response to stress, and, in critical situations, it can activate hazard lights, reduce speed, or initiate emergency braking for enhanced safety.

Mercedes-Benz Energizing Comfort: Although not directly utilizing physiological sensors, this system can interface with wearable devices that track physiological metrics (such as heart rate and stress levels), adjusting the interior settings of the vehicle (lighting, climate, and music) to improve comfort [202].

### 5.3. Semi-Autonomous and Autonomous Driving Support

Audi Traffic Jam Pilot: This system uses interior cameras to monitor driver facial expressions and head positions, assessing their alertness and readiness to take over driving duties from the autonomous system [203].

### 5.4. Future Insights

The development and implementation of DSM systems using physiological sensors present a promising avenue for enhancing road safety. However, challenges such as ensuring the accuracy and reliability of sensor data in dynamic driving environments, addressing privacy concerns related to continuous health monitoring, and integrating these systems seamlessly with existing vehicle architectures remain. Future directions should focus on refining sensor technologies for greater precision, developing advanced algorithms capable of compensating for environmental variables, and establishing robust frameworks for data security and user privacy. Furthermore, the exploration of adaptive systems that can be personalized to individual driver physiological patterns could significantly improve the effectiveness of DSM technologies. Addressing these challenges and following these directions will be crucial in maximizing the full potential of DSM systems to reduce road accidents and improve driver safety.

## 6. Discussion

The increasing importance of monitoring physiological signals in vehicles is transforming road safety and the driving experience. Advances in wearable technology, sensor miniaturization, and wireless connectivity are pivotal in this transformation. These developments, along with the challenges of artifact removal and the integration of drivers’ medical histories, are key areas of focus. The role of comprehensive datasets in shaping robust biosensing models is also crucial. This section aims to shed light on both the current state and future potential of in-vehicle biosensing technologies, highlighting their significant role in fostering safer and more adaptive driving environments.

### 6.1. Advancements in Wearable and In-Vehicle Health Monitoring Technologies

The convergence of wearable technology, sensor miniaturization, wireless connectivity, and real-time monitoring represents a significant advancement in vehicle safety systems. By integrating wearable devices, such as smartwatches and fitness bands, with in-vehicle systems, a more comprehensive overview of the driver’s health is achieved. This synergy enables the seamless transmission of health data to external devices and healthcare systems via wireless connectivity, ensuring that immediate assistance is available in case of a medical emergency while driving. The miniaturization of sensors facilitates their unobtrusive integration into vehicle interiors and wearable devices, enhancing user comfort and encouraging adoption. Real-time health monitoring provides instant feedback on the driver’s physiological state, crucial for preventing accidents related to sudden health issues. Furthermore, personalized health monitoring through customized biosensors tailors this approach to individual needs, offering specific insights for drivers with chronic conditions. Furthermore, measuring physiological signals within a car facilitates the use of biometrics for driver identification [204]. Together, these technologies forge a holistic approach to monitoring driver wellness and alertness, paving the way for safer driving experiences.

### 6.2. Autonomous Driving Integration

Combining continuous health monitoring with autonomous driving technology can greatly improve both road safety and driver well-being. If a driver experiences a sudden health issue—like fainting or a heart problem—the autonomous system can take control of the vehicle and respond by safely pulling over or driving to the nearest medical facility. This kind of response is especially important in semi-autonomous vehicles where the driver is still expected to maintain control at all times.

In addition to emergencies, real-time health data can help the autonomous system to make smarter decisions. For example, if the system detects that the driver is stressed or fatigued, it could adjust the driving style to be smoother or more cautious, or even suggest switching to a higher level of automation if available.

In fully autonomous cars, where no human driving is required, health monitoring may not be as critical for driving control, but it can still be useful. It can track the health of passengers during the ride, especially in long trips or medical transport situations.

### 6.3. Vehicle Vibration and Driver Movement Cancellation

Addressing vehicle vibration and driver movement is crucial in physiological sensing systems for automotive applications. These challenges require advanced cancellation techniques to ensure the accuracy and reliability of the collected data. Among the approaches, adaptive filtering techniques stand out for their effectiveness in mitigating extrinsic artifacts from vehicle dynamics and intrinsic artifacts from driver movements. A key strategy involves the use of multiple sensors, where some are dedicated to measuring physiological signals of interest, while others specifically capture noise, such as vibrations or driver movements. By comparing these inputs, adaptive filtering can dynamically adjust to cancel out the noise, significantly improving data quality. This method is particularly promising due to its ability to adapt in real time to changing conditions and the specific characteristics of the noise [205].

Future efforts should aim at refining these adaptive filtering techniques and exploring their integration across different sensing platforms to enhance data integrity. Standardizing these advanced cancellation methods is also essential to ensure the comparability and reproducibility of the research findings, thus advancing the application of physiological sensing systems in vehicles. This streamlined focus will support the practical deployment of these technologies and make them more robust against the complex dynamics of real-world driving.

### 6.4. Enhanced Data Analysis Through AI

Artificial intelligence significantly enhances vehicle biosensing by enabling more accurate and adaptable real-time health monitoring [206]. These approaches should enable the development of systems that are not only more accurate but also highly personalized and adaptive to individual health patterns over time.

#### 6.4.1. Federated Learning and Privacy

Federated learning stands out by allowing vehicles to collaboratively learn a shared prediction model. This technique keeps all the training data locally in the vehicle, improving privacy and security for sensitive health data. It exemplifies the shift towards decentralized data processing, ensuring that personal health monitoring systems can learn and improve without compromising individual privacy.

#### 6.4.2. Enhanced Learning Approaches

Combining transfer learning and domain adaptation offers a powerful strategy for dealing with the diverse data environments typical in the automotive sector. Transfer learning allows models to apply knowledge gained from one task to another, significantly reducing the need for labeled training data. When paired with domain adaptation, it ensures that models remain effective even when they encounter data with different distributions than those on which they were trained. This is crucial to maintain consistent performance across varied sensor inputs and individual health profiles.

#### 6.4.3. Innovative Model Architectures

The integration of GANs, attention mechanisms with transformer models, and graph neural networks (GNNs) introduces a new level of sophistication to data analysis. GANs help to overcome data scarcity and privacy concerns by generating synthetic biosensor data, allowing for the robust training of models without the need for vast datasets of sensitive information. Attention mechanisms and transformer models, adapted from breakthroughs in natural language processing, are adept at handling time-series biosensor data. They focus on the most relevant information for accurate health status monitoring and anomaly detection. Meanwhile, GNNs excel in analyzing interconnected physiological signals, such as heart rate, blood pressure, and oxygen saturation, modeling the complex interdependencies between different biosignals to offer a comprehensive view of an individual’s health.

#### 6.4.4. Adaptive Learning and Minimal Data Requirements

Reinforcement learning (RL) introduces a dynamic aspect to health monitoring, adjusting the system’s approach in real time based on the current health state or behavior of the occupant. This ensures optimal data collection and processing under varying conditions. Additionally, semi-supervised learning and one-shot learning techniques address the challenges of limited labeled data. Semi-supervised learning leverages both labeled and unlabeled data, offering a practical solution in scenarios where acquiring fully labeled datasets is impractical. One-shot learning, on the other hand, enables the system to recognize patterns and make accurate predictions with minimal prior exposure, crucial for detecting rare health events or adapting to new conditions quickly.

The integration of these AI techniques into vehicle biosensing systems promises improved accuracy, efficiency, and adaptability. This can lead to biosensing systems capable of providing real-time personalized health insights. This not only improves driver safety but also opens the door to a new era of proactive health management for vehicle occupants.

### 6.5. Privacy Concerns and Ethical Considerations

The integration of physiological detection systems into vehicles raises significant privacy and ethical concerns due to the sensitive nature of the collected data. Ensuring the protection of individual privacy necessitates robust data protection measures, including strict encryption protocols and adherence to data protection laws like the GDPR [207]. Transparency in data collection, usage, and access is essential to maintain user trust and ensure informed consent.

Anonymization techniques can help to mitigate privacy risks, but they must be carefully implemented to preserve the utility of data for safety applications. Moreover, ethical guidelines are needed to balance safety enhancements with respect to driver autonomy and to prevent undue stress from continuous monitoring.

### 6.6. Risks of Misclassification and Engineering Responsibility

Beyond concerns of privacy and data security, a critical ethical challenge in physiological driver monitoring systems lies in the impact of incorrect predictions, particularly false negatives and false positives. A false negative, where a health issue goes undetected, may allow an impaired driver to remain in control of the vehicle, increasing the risk of a crash and endangering both the driver and others on the road. In contrast, a false positive, where the system incorrectly flags a healthy driver as unfit, could lead to unnecessary interventions—such as disabling vehicle control—causing frustration, reputational harm, or even legal action.

Engineers and system designers must therefore take proactive responsibility for developing accurate and robust detection algorithms as well as for establishing appropriate thresholds, fallback mechanisms, and human-in-the-loop designs that mitigate these risks.

In addition to system performance metrics, engineers and developers must anticipate the potential social and legal ramifications of misclassification, including biased outcomes across different demographic groups, and ensure that recourse mechanisms are available for affected users. Transparent validation procedures, real-world testing, and inclusive data collection are essential for creating trustworthy systems.

### 6.7. Database and Data Quality Concerns

The significance of large, diverse, and high-quality datasets for in-vehicle biosensing cannot be overstated. These datasets are fundamental for testing various techniques, validating their efficacy, and developing robust models for real-world applications [208]. The key publicly available datasets contributing to this field include MIT DriverDB, which focuses on driver stress under different driving conditions [12]; HciLab, measuring driver workload [209]; StateFarm’s dataset for posture classification [210]; Brain4Car, providing insights into driver–autonomous vehicle interactions [211]; AffectiveRoad, studying driver emotions [212]; and the Distracted Driver Dataset, focusing on manual distractions with global data [153]. The BROOK dataset is another notable addition, encompassing comprehensive driver data in various driving scenarios [208].

However, an often-overlooked aspect in this domain is the driver’s medical history. According to a National Motor Vehicle Crash Causation Survey (NMVCCS), a significant percentage (95%) of accidents caused by medical emergencies involved drivers with a history of medical conditions, such as blackouts, heart attacks, seizures, or diabetic reactions [213]. This highlights the need for biosensing systems that are not just generic but adaptive and capable of adjusting their sensitivity based on individual medical histories. For instance, the EEG readings for an epileptic patient differ significantly from those of a non-epileptic individual. Similarly, chronic abnormalities in ECG signals—common in certain individuals—may not impair driving ability but still deviate from the ’normal’ signal pattern used for model training. Thus, detection systems must be able to adapt to such individual circumstances to avoid false alarms or misclassifications. Therefore, vehicle physiological monitoring systems must include flexible and adaptive settings to accurately accommodate drivers with diverse medical histories.

Nonetheless, these atypical conditions are relatively rare, raising the question of how to acquire sufficient data for training robust models in such edge cases. Potential solutions include synthetic data generation, domain adaptation techniques, or collaboration with medical institutions to access anonymized clinical datasets relevant to driving contexts.

It is imperative that future research and development in this field focus on creating driver-dependent algorithms with parameters that can be customized and updated according to each driver’s specific health conditions and driving patterns. While the current research largely concentrates on detecting issues, there is a pressing need to shift towards predictive models that can foresee and mitigate risks associated with known medical conditions. This approach could significantly reduce the incidence of accidents caused by medical emergencies while driving.

## 7. Conclusions

This review highlights the burgeoning potential of in-vehicle biosensing, showcasing a diverse landscape of sensing modalities, current capabilities, and promising applications. Although the field is still maturing, several technologies are already demonstrating promising results in non-invasive driver monitoring with minimal disruption to the driving experience. Advancements in in-cabin systems that assess driver alertness and attention, as well as physiological monitoring techniques offering insights into driver health, are particularly noteworthy. However, ongoing research and development are needed to enhance the accuracy, reliability, and user comfort of these systems.

Despite these advances, biosensing in vehicles remains a challenging issue for automobile manufacturers and suppliers. Looking ahead, the next 10 years are expected to feature significant progress. The future trends will likely focus on integrating artificial intelligence and machine learning to improve monitoring precision and responsiveness. Enhanced driver monitoring systems that assess alertness, fatigue, and cognitive load using eye-tracking, facial recognition, and heart rate variability sensors are anticipated. Real-time health monitoring technologies tracking vital signs like heart rate and respiration rate to detect health issues such as stress and fatigue are also on the horizon.

The development of multimodal sensing platforms combining data from various sensors, such as ECGs and PPGs, will provide a more comprehensive understanding of the driver’s state. These advancements will enable smart interventions, where systems not only monitor but also respond to the driver’s condition by adjusting the vehicle’s environment or alerting the driver to take a break if signs of fatigue are detected. Integration with autonomous driving technologies is another promising area, where biosensing systems could help to determine when to transition control between the vehicle and the driver based on the driver’s readiness and condition.

While challenges remain, the possibility of seeing advanced in-vehicle biosensing technologies in off-the-shelf vehicles in the near future is promising. As these technologies advance, they hold the potential to significantly enhance road safety and driver wellness, paving the way for smarter, more responsive vehicles.

## Figures and Tables

**Table 2:** Overview of the applications of in-vehicle biosensing.

Application	Notable Studies	Methodology	Results
Detection of Heart Attack	M.E.H. Chowdhury et al. (2019) [133]	Developed an SVM classifier for ECG classification using data from 28 individuals from the MIT-BIH ST Change Database.	The classifier demonstrated a 97.4% accuracy rate in identifying ST-elevation myocardial infarctions and a 96.3% accuracy rate for non-ST-elevation myocardial infarctions.
Detection of Fatigue and Drowsiness	F. Wang et al. (2023) [134]	Utilized elastic dry electrodes for ECG signal acquisition from the palm, introducing a periodic man–machine interaction mode (PMIM) to evaluate and mitigate driver fatigue. Sixteen healthy subjects underwent a simulated driving experiment on a monotonous highway in two modes: normal and PMIM, to induce driving fatigue.	Demonstrated effective fatigue reduction with fatigue recognition accuracies ranging between 94% and 99% in various experimental conditions.
	J. Gwak et al. (2020) [135]	Utilized EEG, ECG, and behavioral indices to classify driver states using a random forest. The method was tested in a study involving 16 participants in a monotonous driving simulation aimed at inducing drowsiness.	Achieved 82.4% accuracy in distinguishing between alert and slightly drowsy states and 95.4% accuracy between alert and moderately drowsy states.
Detection of Stress and Emotional Diriving	D.S. Lee et al. (2016) [136]	Utilized a wearable glove with fingertip PPG sensing to monitor stress and emotional states in real time. Twenty-eight subjects were requested to perform three different driving sessions with random scenarios generated while performing various driving maneuvers to assess the dynamics of mental workloads.	Achieved 95% accuracy in identifying stressful driving conditions.
	Z. Halim & M. Rehan. (2020) [137]	EEG sensors were utilized to capture brain activity, focusing on stress detection. The SVM algorithm was used to analyze the collected data collected from a group of 50 drivers.	Achieved 97.95% accuracy, showcasing superior performance over alternative classifiers.
Detection of Dangerous Driving	F. Yan et al. (2019) [138]	EEG and driving data were analyzed using K-means clustering and SVM modeling to classify driving styles. Experiments were performed on 23 participants under 75 driving tasks.	Achieved 80% accuracy with SVM, distinguishing conservative and aggressive driving styles by their unique EEG patterns.
Detection of DriverDistraction	O. Dehzangi et al. (2018) [17])	Engaged 10 driver subjects in real driving experiments, measuring phasic EDA via a wristband wearable. Applied continuous decomposition analysis for signal processing and SVM with recursive feature elimination for feature selection and classification.	Achieved a cross-validation accuracy of 94.81% with all features and 93.01% with a reduced feature set, demonstrating effective distraction identification.
Detection of Impaired Driving	C. K. Wu et al. (2016) [139]	Designed an ECG-based Drunk Driving Detection (DDD) system using a classifier with weighted kernel functions. The DDD system was evaluated on 50 volunteers in a stationary car under normal and intoxicated conditions.	Achieved an accuracy of 87.52%, outperforming conventional methods by 11%.

**Table 3:** Overview of machine learning for in-vehicle biosensing.

Algorithm Category	Description and Examples	Requirements, Assumptions, and Applications	Examples of Applications
Statistical Machine Learning	Involves models that infer relationships from statistical analysis. **Examples**: SVM, k-nearest neighbor (KNN), Bayesian networks.	Assumes certain data distributions; requires extensive data preprocessing and feature engineering; ideal for unimodal biosensing with custom features, especially when minimizing overfitting is crucial.	M. Choi et al. (2020) introduced a fuzzy SVM for personalized fatigue, drowsiness, and stress monitoring, analyzing PPG, GSR, temperature, and motion data. It achieved 92% accuracy and outperformed traditional SVM.
Ensemble Methods	Techniques that combine predictions from multiple models to improve accuracy. **Examples**: random forest, gradient boosting.	Assume that combining multiple models increases accuracy; ideal for biosensing applications with user-defined features, improving accuracy and reducing overfitting via diverse models.	N. Du et al. (2022) [162] designed a random forest to predict drivers’ takeover performance in conditionally automated driving by analyzing heart rate, EDA, eye-tracking metrics, and driving conditions. Tested on two indidivuals and achieved an accuracy of 84.3% using a 3-s prediction window.
Neural Networks	Comprises algorithms modeled on the human brain’s architecture, suitable for capturing complex and nonlinear patterns. **Examples**: multi-layer perceptron (MLP), RNN (LSTM, Gated recurrent unit—GRU), CNN.	There is no specific assumption; require large amounts of data and high processing power; suitable for processing and learning from large, intricate, and unprocessed datasets.	M Peivandi et al. (2023) [163] introduced a novel approach for multi-level driver fatigue detection from ECG, EEG, EMG, and respiratory signal utilizing CNNs. Tested on 20 students and achieved accuracies ranging from 89% to 96%.
Advanced Deep Learning Models	Focuses on state-of-the-art neural network architectures for complex pattern recognition and generation. **Examples**: Transformers (for sequence modeling tasks), **GAN** (for generating new data instances).	Assume complex relationships in data; require substantial computational resources and diverse datasets; effective for data generation (GANs) and recognizing long-term patterns in physiological data (Transformers), delivering unparalleled performance.	L. Mou et al. (2023) [164] implemented a dual-channel model combining CNN and Transformer for driver distraction detection on a multimodal dataset. Tested on 68 individuals and achieved a high accuracy of 99%.
Hybrid Methods	Integrates various machine learning techniques to exploit their combined strengths. **Example**: Combining CNN with RNN for complex tasks.	Integrates diverse neural architectures; demands innovative design and substantial data; efficient in analyzing multimodal and complex physiological data.	D. Zhou et al. (2023) [165] developed a multimodal fusion framework combining the driver’s voice, facial image, and video sequence data to recognize a range of emotions using a hybrid CNN + Bi-LSTM + attention model. Tested on 123 individuals and achieved a recognition rate of 85.5%.
Feature Selection Methods	Identifies the most relevant features for the model to improve performance and reduce overfitting. **Examples**: Recursive Feature Elimination (RFE), Principal Component Analysis (PCA).	Assumes specific relationships among features; simplifies data by reducing dimensions; suitable for improving model efficiency and interpretability of complex biosensing data.	Y. Huang et al. (2022) [166] combined PCA and MLP to identify driving drowsiness using PPG, EDA, and respiration data. Tested on nine individuals and achieved 97% accuracy.

**Table 4:** Industrial driver status monitoring prototypes.

	Prototype	Description	Sensors
Fatigue	Caterpillar Driver Safety System (DSS) [189]	It uses in-cab cameras and sensors to monitor drivers for fatigue and distraction, providing real-time alerts and data to a central system. The system employs facial recognition cameras, seat sensors, steering wheel sensors, and GPS to analyze driver behavior and alertness levels.	In-cab cameras, facial recognition cameras, seat sensors, steering wheel sensors, GPS
	SmartCap [190]	The SmartCap is a wearable cap with EEG sensors that monitor brain activity and detect fatigue levels. It provides real-time alerts and integrates with fleet management systems to improve driver safety and productivity.	EEG sensors
	Guardian by Seeing Machines [191]	Guardian uses a dash-mounted camera and sensors to track drivers’ head and eye movements. It detects signs of fatigue and distraction, providing real-time alerts and detailed data analysis for proactive safety measures.	Dash-mounted camera, infrared sensors, head and eye tracking sensors
	Optalert’s Eagle [192]	Eagle consists of glasses equipped with an infrared sensor and IR LED that continuously monitor eyelid movements and blink patterns. It provides real-time feedback via the Johns Drowsiness Score and integrates with fleet management systems to enhance driver alertness and safety.	Infrared sensor and IR LED
Drowsiness	Bosch Driver Drowsiness Detection System [193]	Bosch’s system uses steering angle sensors and vehicle speed data to assess driver drowsiness. It alerts the driver with audio and visual signals, helping to prevent accidents caused by reduced alertness.	Steering angle sensors, vehicle speed sensors
	Denso’s Driver Status Monitor [194]	Denso’s system uses a camera and infrared sensors to monitor the driver’s face for signs of drowsiness and inattention. It integrates with other vehicle safety systems to provide timely alerts and ensure driver attentiveness.	Camera, infrared sensors
	Mercedes-Benz ATTENTION ASSIST [195]	ATTENTION ASSIST monitors steering movements and driving behavior to detect signs of driver drowsiness. It alerts the driver with visual and acoustic signals, promoting safer driving habits and reducing fatigue-related accidents.	Steering movement sensors, vehicle behavior sensors, GPS
Distraction	Nauto Driver Safety System [196]	Nauto combines AI-powered cameras and sensors to monitor driver behavior, including distractions and drowsiness. It delivers real-time alerts and comprehensive fleet management reports for proactive safety measures.	AI-powered cameras, infrared sensors, GPS
	Tesla Driver Engagement Monitoring [197]	Tesla’s system uses in-cabin cameras and AI-powered sensors to monitor driver attention and engagement. It ensures drivers remain focused on the road and alert to interact with semi-autonomous driving features effectively.	In-cabin cameras, AI-powered sensors, GPS
	Volvo Intoxication and Distraction System [198]	Volvo’s system uses in-car cameras and sensors to monitor signs of driver intoxication and distraction. It automatically intervenes if necessary to prevent accidents and ensure safe driving conditions.	In-car cameras, alcohol sensors, GPS
Health Metrics	Toyota’s Steering Wheel with Heart Rate Sensors [199]	Toyota integrates heart rate sensors into the steering wheel to monitor the driver’s physical condition. It detects signs of stress or health issues and provides alerts to maintain driver well-being and safety.	Steering wheel heart rate sensors
	Ford’s Health-Focused Technologies [200]	Ford’s system includes wearable device integration and in-car sensors to monitor the driver’s health metrics such as heart rate and glucose levels. It provides alerts and feedback to enhance driver well-being and safety during journeys.	Wearable device integration, in-car sensors (heart rate sensors, glucose sensors), GPS
	BMW and Wearable Device Interface [201]	BMW integrates wearable devices with vehicle systems to monitor driver health metrics such as heart rate and stress levels. It provides real-time feedback and adjusts car settings for safety and comfort during driving.	Wearable devices (smartwatches, fitness trackers), vehicle system integration (CAN bus), GPS
Other	Mercedes-Benz Energizing Comfort [202]	Mercedes-Benz uses in-car climate control, ambient lighting, and music to improve driver well-being and reduce fatigue during long journeys. The system adjusts environmental factors to enhance driver comfort and alertness.	In-car climate control sensors, ambient lighting sensors, audio sensors, GPS
	Audi TrafficJam Pilot [203]	Audi’s system utilizes a combination of cameras, sensors, and AI to monitor driver attention during traffic jams. It ensures the driver is ready to take over when necessary, enhancing safety and convenience in congested traffic conditions.	Cameras, radar sensors, ultrasonic sensors, AI

## Data Availability

No new data were created or analyzed in this study.
